# Gene Profiling of the Ascorbate Oxidase Family Genes under Osmotic and Cold Stress Reveals the Role of AnAO5 in Cold Adaptation in *Ammopiptanthus nanus*

**DOI:** 10.3390/plants12030677

**Published:** 2023-02-03

**Authors:** Ming Zhu, Qi Liu, Fuyu Liu, Lamei Zheng, Jie Bing, Yijun Zhou, Fei Gao

**Affiliations:** 1 Key Laboratory of Mass Spectrometry Imaging and Metabolomics, Minzu University of China, National Ethnic Affairs Commission, Beijing 100081, China; 2 Key Laboratory of Ecology and Environment in Minority Areas, Minzu University of China, National Ethnic Affairs Commission, Beijing 100081, China; 3 College of Life and Environmental Sciences, Minzu University of China, Beijing 100081, China; 4 College of Life Sciences, Beijing Normal University, Beijing 100080, China

**Keywords:** ascorbate oxidase, *Ammopiptanthus nanus*, osmotic stress, cold stress, gene family

## Abstract

The uplift of the Qinghai Tibet Plateau has led to a drastic change in the climate in Central Asia, from warm and rainy, to dry and less rainfall. *Ammopiptanthus nanus*, a rare evergreen broad-leaved shrub distributed in the temperate desert region of Central Asia, has survived the drastic climate change in Central Asia caused by the uplift of the Qinghai-Tibet Plateau. Ascorbate oxidase (AO) regulates the redox status of the apoplast by catalyzing the oxidation of ascorbate acid to dehydroascorbic acid, and plays a key role in the adaptation of plants to environmental changes. Analyzing the evolution, environmental response, and biological functions of the AO family of *A. nanus* is helpful for understanding how plant genome evolution responds to climate change in Central Asia. A total of 16 AOs were identified in *A. nanus*, all of which contained the ascorbate oxidase domain, most of which contained transmembrane domain, and many were predicted to be localized in the apoplast. Segmental duplication and tandem duplication are the main factors driving the gene amplification of the AO gene family in *A. nanus*. Gene expression analysis based on transcriptome data and fluorescence quantitative PCR, as well as enzyme activity measurements, showed that the expression levels of AO genes and total enzyme activity decreased under short-term osmotic stress and low-temperature stress, but the expression of some AO genes (*AnAO5*, *AnAO13*, and *AnAO16*) and total enzyme activity increased under 7 days of cold stress. *AnAO5* and *AnAO11* are targeted by *miR4415*. Further functional studies on AnAO5 showed that AnAO5 protein was localized in the apoplast. The expression of *AnAO5* in yeast cells and the transient expression in tobacco enhanced the tolerance of yeast and tobacco to low-temperature stress, and the overexpression of *AnAO5* enhanced the tolerance of Arabidopsis seedlings to cold stress. Our research provides important data for understanding the role of AOs in plant adaptation to environmental change.

## 1. Introduction

The uplift of the Qinghai-Tibet Plateau and the rise of the Himalayas have had a profound impact on the climate in Central Asia [1,2]. Research has shown that the climate in Central Asia was once warm and rainy [3], and Fossil evidence suggests that the plants distributed in the region were mainly evergreen broad-leaved plants in the early Holocene and middle Holocene [4,5]. With the multiple uplifts of the Qinghai-Tibet Plateau and the weakening of the Asian ocean monsoon, the climate in Central Asia began to become drier and cooler in the late Holocene [6]. The original broad-leaved forest gradually turned into temperate steppe and gradually evolved into the current desert vegetation [7]. *A. nanus* is a rare evergreen broad-leaved shrub grown in the desert area of Central Asia, and is a relict plant in the ancient Mediterranean region [8,9]. *A. nanus* has survived the drastic climate change in Central Asia caused by the uplift of the Qinghai-Tibet Plateau. Analyzing the evolution of the genome of *A. nanus* is helpful to understanding the response of plant evolution to climate change and the adaptive evolution of plants.

The apoplast is the first barrier between cells and the external environment, and an important part allowing plants to sense environmental changes [10,11]. ROS is one of the key signal molecules for plants to perceive changes in the external environment and transmit the signal to cells [12,13,14]. Environmental factors, including abiotic stress such as drought, high temperature, and low temperature, and biotic stress, including insect bites and pathogenic fungal invasion, are transmitted to cells through ROS signals generated in the apoplast and then producing cellular effects [15,16,17]. Ascorbate oxidase (AO, EC 1.10.3.3) belongs to the family of copper oxidase and is widely found in the plant kingdom [18]. AO is an apoplast-localized enzyme that plays an important role in regulating reactive oxygen species (ROS) levels in the apoplast by catalyzing the oxidation of ascorbate acid (ASA) to dehydroascorbic acid (DHA), and it is considered as one of the main factors affecting the redox balance of the apoplast [19,20,21]. Analyzing the structure and evolution of the AO gene family and its response to environmental stresses such as drought and low temperature stress will help to understand the response of plants to the impact of the Qinghai-Tibet Plateau uplift and the climate in Central Asia.

As early as 1931, after the discovery of AO in cabbage leaves [22], a number of AOs were found in several cucurbitaceae plants. Recent studies have shown that AO is involved in regulation of multiple biological processes, including plant abiotic and biotic stress response [23,24], growth and development [25,26,27], and flowering induction [28]. One study showed that the enhancement of AO activity in transgenic tobacco had little effect on the overall ascorbate acid pool in the whole leaf, but significantly affected the ascorbate acid pool in the apoplast and changed the redox state of the ascorbate acid pool in the apoplast [29,30], supporting that the redox state of plastids is largely determined by AO [31]. The increase of AO activity led to greater ROS accumulation, which enhanced the plant’s defense ability against a virus in rice [32]. Another AO was shown to regulate the tolerance to salt stress in rice [33]. These works advanced the understanding of the biological functions of plant AOs. However, the previous studies mainly focused on the function of a single AO [34,35,36], and a genome-wide analysis of the structure, evolution, and response to abiotic stress of AO is still lacking.

In the present study, whole-genome identification of the AO family of *A. nanus* was conducted, and the chromosome distribution, phylogenetic relationships, gene duplication events, expression profiling, and AO activity of the AO gene family members under drought and cold stress in *A. nanus* were analyzed. The biological function of AnAO5 was further investigated through expression in yeast and tobacco seedlings. We also overexpressed AnAO5 in Arabidopsis, to evaluate its role in cold stress response. This study will provide important data for understanding the biological function of AOs in plant adaptation to environmental changes.

## 2. Results

### 2.1. Genome-Wide Identification of AOs in A. nanus

**Figure 1:**
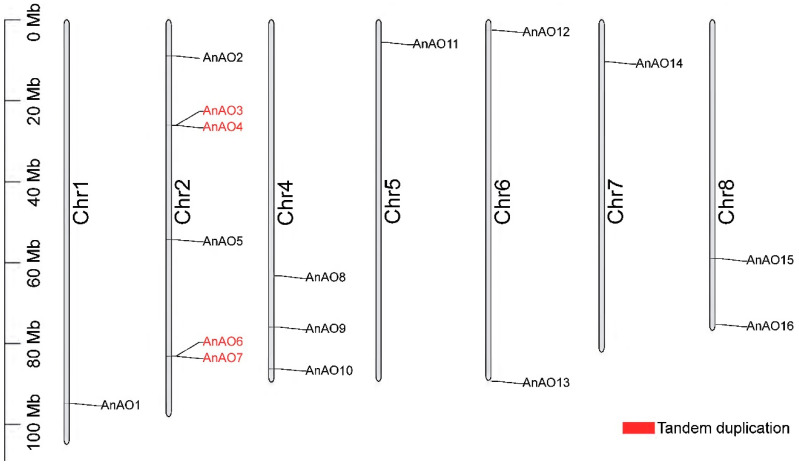
The distribution of *AnAO* genes in the chromosomes of *A. nanus*. Represented as tandem duplication.

A total of 16 AOs were identified from the genome of *A. nanus* (Table 1). The amino acid length of AO family proteins is similar, ranging from 517 to 577 amino acids. The largest *A. nanus* AO (AnAOs) is AnAO16, and the smallest is AnAO15. Most AnAOs are alkaline proteins, except AnAO9, which is an acidic protein. All AnAOs were hydrophilic proteins and up to 68.75% of AnAOs were stable proteins (II < 40). A subcellular localization analysis showed that a considerable number of AOs were localized in the extracellular matrix or vacuole. AO gene family members are distributed on seven of the nine chromosomes of *A. nanus*. There are six AO genes distributed in chromosome 2, while no AO genes were detected on chromosomes 3 and 9 (Figure 1).

### 2.2. Structural Analysis of the AO Family in A. naus

**Figure 2:**
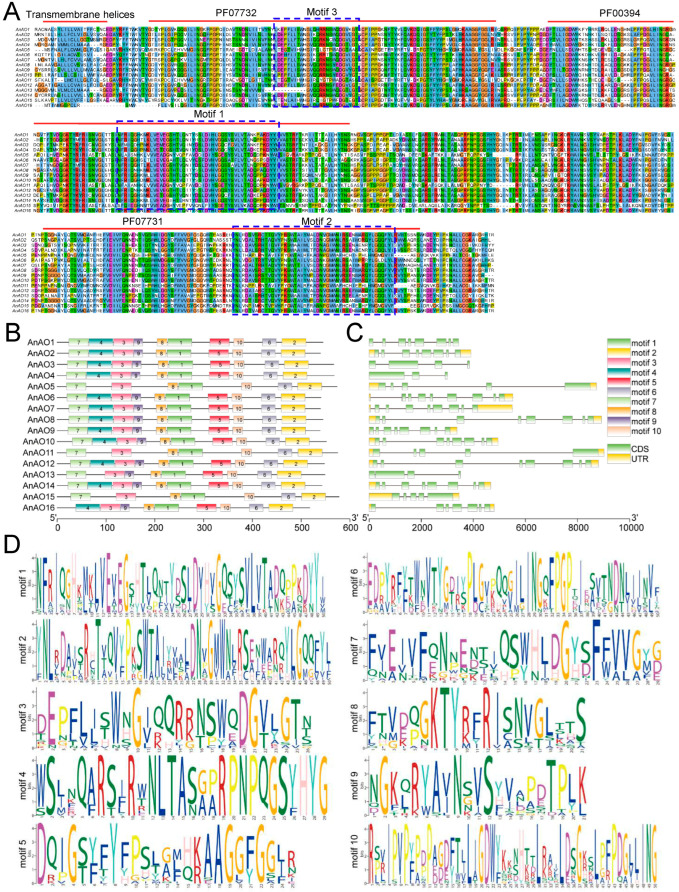
Structural analysis of the AnAOs of *A. nanus*. (**A**) Multiple sequence alignment analysis of AOs in *A. nanus*. (**B**) The conserved motifs of AO gene family in *A. nanus*. All motifs were predicted using the MEME database. The rectangular box represented the motif, and different colors represent different motifs. (**C**). The intro-exon structures of AO family genes. Black lines represent introns, yellow rectangles represent CDS, and green rectangles represent untranslated regions (UTRs). (**D**) Motif sequence logo graph. The relative size of the letters represents their frequency in the sequence. The height of each letter is proportional to the frequency of occurrence of the corresponding base at that position.

Multiple sequence alignment analysis showed that all AnAOs contained a typical ascorbate oxidase domain, and most AOs contained a transmembrane domain at the N-terminal, indicating that most AOs were secretory proteins (Figure 2A). MEME analysis showed that all AnAO proteins contained motif1, motif2, and motif3, which overlaped with PF00394, PF07731, and PF07732, respectively. Motif4, motif5, and motif6 were present in the majority of AnAOs, but they were not detected in AnAO5, AnAO11, and AnAO15 (Figure 2B).

The gene locus lengths of AnAO5, AnAO8, AnAO11, and AnAO12 were significantly longer than those of the other AnAOs, and the numbers of introns of AnAO loci were different. The gene loci of AnAO1, AnAO2, AnAO6, and AnAO7 contained up to seven introns, while only two introns were present in the gene locus of AnAO4 (Figure 2C,D).

### 2.3. Phylogenetics and Gene Duplication and Divergence of the AO Family in A. nanus

**Figure 3:**
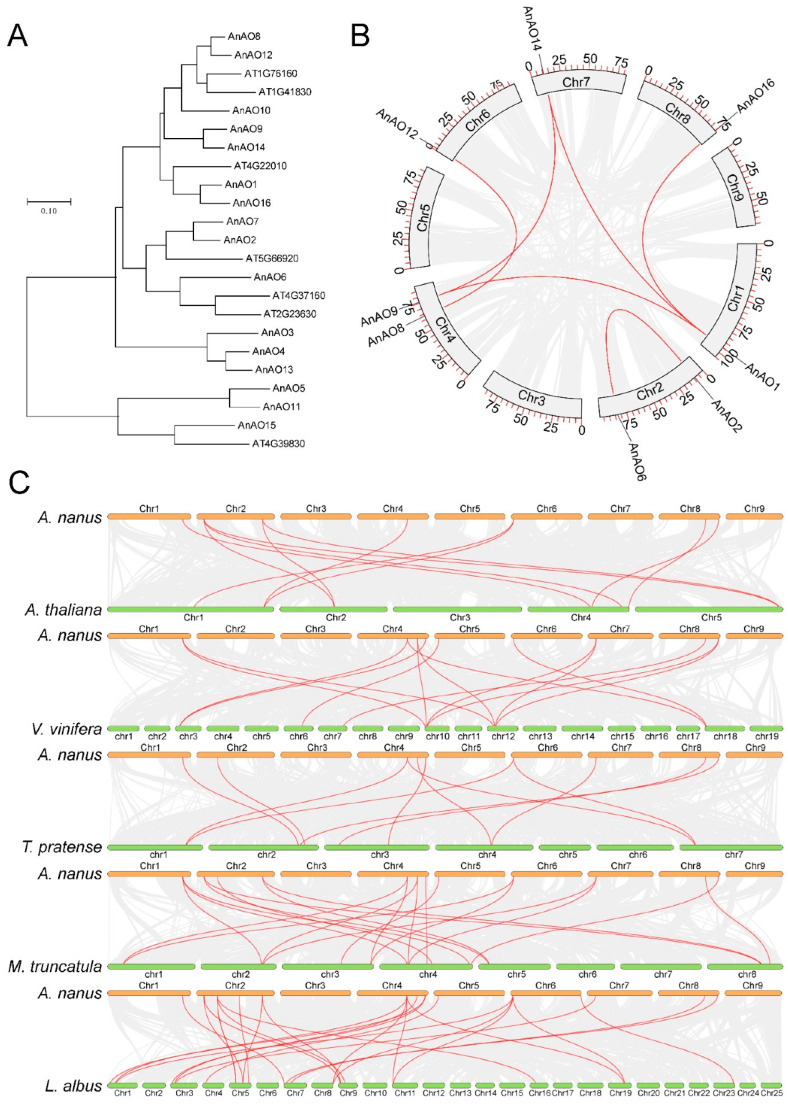
Phylogenetics, and gene duplication and divergence of the AO family in *A. nanus*. (**A**) The phylogenetic tree of the AnAO family. Different colors represent different plant species, red is *A. nanus* and black is *A. thaliana*. (**B**) The distribution of segmental duplication genes of AnAO on the chromosome of *A. nanus*. (**C**) Homologous gene pairs between *A. nanus* and *V. vinifera*, *A. thaliana*, *M. truncatula*, *T. pratense*, and *L. albus*.

To analyze the evolutionary relationship of the AO gene family in *A. nanus*, a phylogenetic tree was constructed using the amino acid sequences of AOs in *A. nanus* and *Arabidopsis thaliana*. Phylogenetic analysis showed that all AnAOs were clustered into four clades. AnAO5, AnAO11, and AnAO15 formed one branch that diverged earliest, and AnAO3, AnAO4, and AnAO13 formed another branch that diverged later than the branch consisting of AnAO5, AnAO11, and AnAO15 (Figure 3A).

A total of eight AnAOs underwent segmental duplication and four AnAOs underwent tandem duplication, indicating segmental duplication and tandem duplication were the two main modes of duplication of the AO family in *A. nanus* (Figure 1 and Figure 3B). In order to reveal the evolution history of AO members, we drew Ks distribution curves of the AO genes that underwent segmental duplication (Figure S1) and found that four pairs of AO genes that had undergone segmental duplication (i.e., AnAO1–AnAO16, AnAO2–AnAO6, AnAO8–AnAO12, and AnAO9–AnAO14.) were amplified in a whole genome duplication (WGD) event in approximately 85.37 Mya, while the other two pairs of AO genes that had undergone segmental duplication (AnAO1–AnAO14 and AnAO1–AnAO9) were duplicated in an earlier WGD event. Synteny analysis showed that there were 6, 7, 7, 11, and 18 orthologous gene pairs between *A. nanus* and *Vinifera vinifera*, *A. thaliana*, *Medicago truncatula*, *Trifolium pratense*, and *Lupinus albus*, respectively (Figure 3C).

To investigate the adaptive evolution of the AO gene family in *A. nanus*, a positive selection analysis was performed for all paralogous gene pairs. The Ka/Ks value of the genes that had undergone negative selection was less than 1, and the Ka/Ks value of the genes that had undergone positive selection was higher than 1. All Ka/Ks values for AnAO paralogous pairs were less than 1, indicating that all AnAO genes had undergone purifying selection (Table 2).

### 2.4. Prediction of the Cis-Acting Elements in Promoter Regions of the AO Family Genes in A. nanus

**Figure 4:**
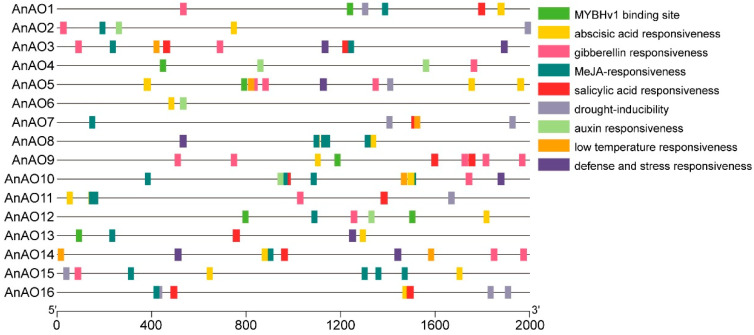
Distribution of the predicted cis-acting elements that were involved in abiotic stress response and hormone response in the promoter region of the AO family genes in *A. nanus*. Different color blocks represent different types of cis-acting elements.

To understand the potential expression patterns of AnAO genes, the cis-acting elements involved in abiotic stress response and hormone response in the promoter regions of AnAO family genes were predicted (Figure 4). The predicted cis-acting elements in the promoter regions of AnAO genes were involved in the response to multiple environmental stresses, such as low-temperature and drought stress, and hormones such as abscisic acid, gibberellin, salicylic acid, and auxin. There were 13 AnAO genes carrying the cis-acting elements involved in ABA response, including *AnAO5*, *AnAO1*, and *AnAO2*. A total of seven *AnAO* genes carried the cis-acting elements involved in drought stress response, including *AnAO11*, *AnAO15*, and *AnAO5*. There were five *AnAO* genes containing the cis-acting elements involved in low temperature response, including *AnAO3*, *AnAO5*, and *AnAO7*. Some *AnAO* genes contained more cis-acting elements involved in abiotic stress response and hormone response, including *AnAO3*, *AnAO5*, *AnAO9*, *AnAO14*, and *AnAO10*, while only two were detected in the promoter region of *AnAO6*.

### 2.5. AnAO5 and AnAO11 Were Targeted by miR4415

**Figure 5:**
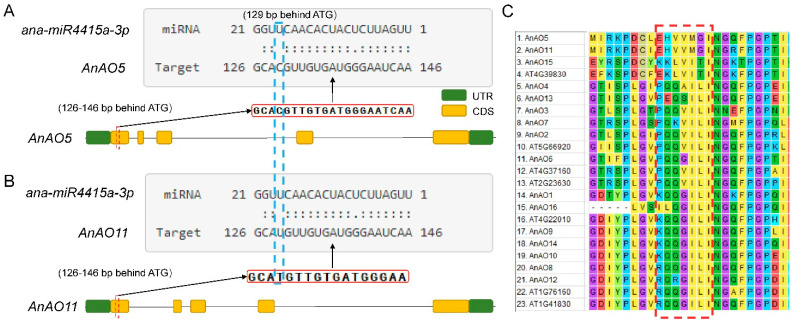
Binding sites of ana-miR4415a-3p on AnAO genes, as predicted by psRNAtarget. (**A**) AnAO5 was targeted by ana-miR4415a-3p. (**B**) AnAO11 was targeted by ana-miR4415a-3p. (**C**) Multi-sequence alignment of AnAOs protein and AtAOs protein depicting the difference in the amino acid sequence of AnAOs corresponding to the binding sites of ana-miR4415a-3p. Red dotted line box represents the amino acid sequence encoding the nucleotides at the binding site of ana-miR4415a-3p.

Previous studies showed that the AnAO5 gene was targeted by ana-miR4415a-3p, a leguminous species-specific miRNA, suggesting that other AnAO genes may also be the target genes of ana-miR4415a-3p. Target prediction using the software psRNAtarget and TargetFinder was conducted, to identify other AnAO genes targeted by ana-miR4415a-3p, and AnAO5 and AnAO11 were found to be targets of ana-miR4415a-3p (Figure 5A,B).

The predicted binding sites of ana-miR4415a-3p were located in the coding regions of AnAO5 and AnAO11 genes (126–146 bp), corresponding to the amino acid sequence in the conserved ascorbate oxidase domain PF07732 (Figure 2A). Multiple sequence alignment of the amino acid sequence corresponding to the binding site of ana-miR4415a-3p showed that the corresponding amino acid sequence on other AnAO proteins was significantly different from that of AnAO5 and AnaAO11 proteins (Figure 5C).

### 2.6. Expression Patterns of A. nanus AO Genes under Osmotic and Low-Temperature Stresses

**Figure 6:**
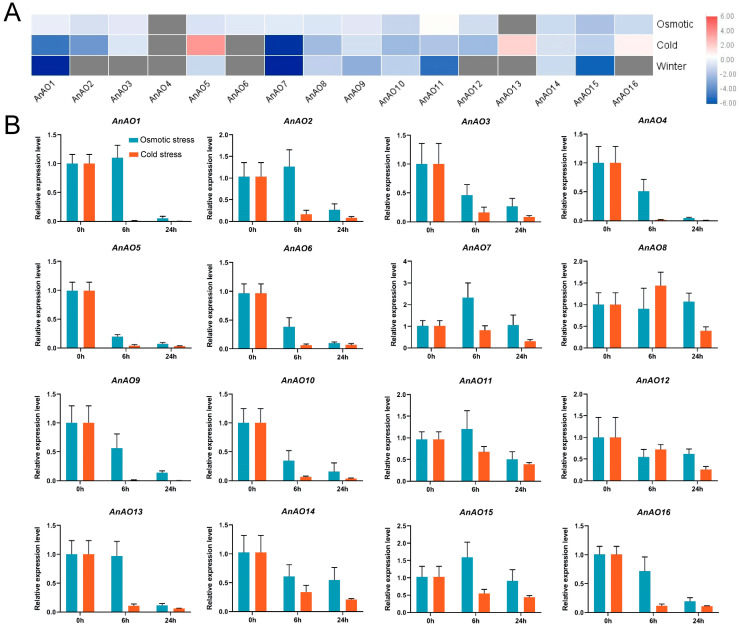
Expression analysis of AnAOs based on transcriptome data and qRT-PCR analysis. (**A**) Expression patterns of AnAO genes under osmotic and cold stress for 7 d and during winter based on transcriptome data. (**B**) Expression patterns of AnAO genes under short-term osmotic stress and cold stress based on qRT-PCR analysis. Each experiment was performed in three independent biological replicates, and the error bar shows the SD (*n* = 3).

To further evaluate the potential functions of the AO family genes, especially their involvement in osmotic and low-temperature stress responses, the expression patterns of AO family genes were analyzed using publicly available RNA-seq data of *A. nanus* and qRT-PCR analyses. The transcriptome data showed that most AnAO genes were downregulated under osmotic stress and cold stress and in winter (compared with spring), except three *AnAO* genes, i.e., *AnAO5*, *AnAO13*, and *AnAO16*, which were upregulated under cold stress (Figure 6A).

qRT-PCR analysis showed that the majority of *AnAO* genes were downregulated under short-term osmotic stress, and the expression levels of several *AnAO* genes, including *AnAO7*, *AnAO8*, and *AnAO15*, did not change significantly (Figure 6B). Under cold stress, the expression levels of all *AnAO* genes were downregulated significantly (Figure 6B).

### 2.7. AO Activity of A. nanus Leaves under Osmotic Stress and Low-Temperature Stress

**Figure 7:**
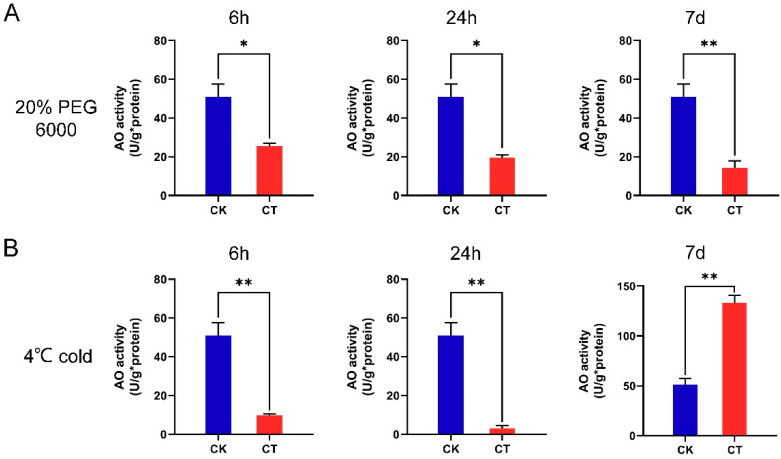
AO activity measurement of the *A. nanus* leaves under osmotic and cold stress. (**A**) AO activities of leaves of *A. nanus* under osmotic stress. (**B**) AO activities of leaves of *A. nanus* under cold stress. Values are expressed as means ± SD (*n* = 3), and statistically significant differences were evaluated using Student’s t-test, * *p* < 0.05, ** *p* < 0.01.

Under osmotic stress and cold stress, the change of AO gene expression may affect the activity of AO in the leaves of *A. nanus.* In order to analyze the effect of osmotic stress and cold stress on AO activity, the AO activities in the leaves of *A. nanus* were measured before and after osmotic stress and cold stress treatment (Figure 7). The measurement of AO activity showed that under osmotic stress, the AO activity in leaves of *A. nanus* decreased significantly at 6 h, 24 h, and 7 d timepoints. Under cold stress, the activity of AO decreased significantly at 6 h and 24 h, but increased significantly at the 7 d timepoint.

### 2.8. Phylogenetic and Sequence Analyses of AnAO5

The expression of AnAO5 increased under 7 days of low temperature stress, which is consistent with the change of AO enzyme activity under cold stress. AnAO5 gene may play an important role in low-temperature adaptation, and further functional investigations were carried out to confirm the function of the AnAO5 gene.

**Figure 8:**
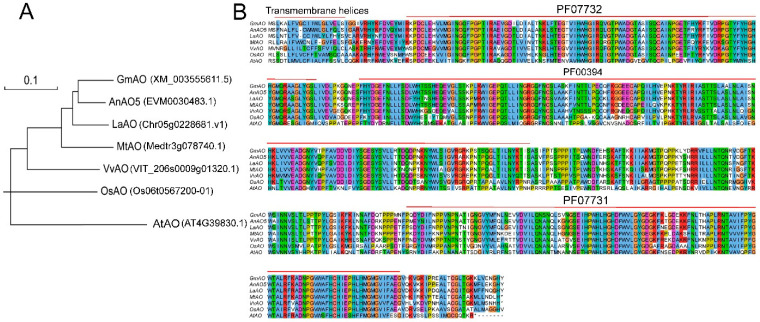
Phylogenetic analyses of AnAO5 and its orthologs. (**A**) The phylogenetic tree of AnAO5 and its orthologs. (**B**) Multiple sequence alignment of AnAO5 and its orthologs.

The total length of the AnAO5 locus is 8727 bp, including an open reading frame of 1719 bp, which encoded 573 amino acids. The amino acid sequence of AnAO5 and the homologous proteins in *V. vinifera*, *A. thaliana*, *Oryza sativa*, *M. truncatula*, *Glycine max*, and *L. albus* were used to construct the phylogenetic tree. The relationship between AO proteins from different plants revealed by the phylogenetic tree is generally consistent with the evolutionary relationship of these plant species, and the AOs of all leguminous plants are clustered into a clade (Figure 8A). Multiple sequence alignment analysis showed that the amino acid sequence of AnAO5 was highly conserved, containing the three ascorbate oxidase domain models (PF00394, PF07731, and PF07732) in the Pfam database, and carrying a transmembrane domain at the N terminal (Figure 8B).

### 2.9. Expression Patterns of AnAO5 in Different Tissues and under Osmotic and Cold Stress

**Figure 9:**
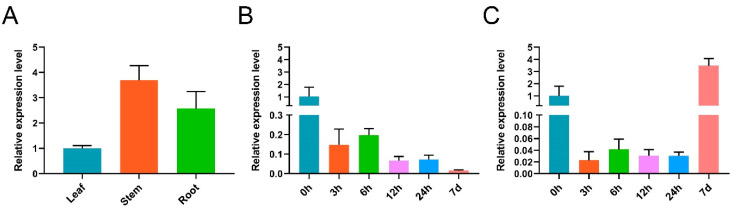
Expression analysis of *AnAO5* gene using qRT-PCR analysis. (**A**) Expression of *AnAO5* gene in leaves, stems, and roots of *A. nanus*. (**B**) Expression patterns of *AnAO5* gene under osmotic stress. (**C**) Expression patterns of *AnAO5* gene under cold stress. *A. nanus eIF1* was used as the internal control. Each experiment was performed in three independent biological replicates, and the error bar showed SD (*n* = 3).

To study the tissue expression and the abiotic stress response of the AnAO5 gene, the gene expression of the AnAO5 gene in roots, stems, and leaves, and before and 3 h, 6 h, 12 h, 24 h, and 7 d after osmotic stress and cold stress were quantitated using qRT-PCR analysis. The expression of AnAO5 was detected in the leaves, stems, and roots of *A. nanus*, and the expression in stems was higher than that in leaves and roots (Figure 9A). Under osmotic stress, the expression of AnAO5 was significantly decreased at 3 h, 6 h, 12 h, 24 h, and 7 d, and the expression was the lowest at 7 d of osmotic stress (Figure 9B). Under cold stress, the expression of AnAO5 decreased significantly at 3 h, 6 h, 12 h, and 24 h, but increased significantly at 7 d (Figure 9C).

### 2.10. The AnAO5 Protein Was Localized in Apoplast

**Figure 10:**
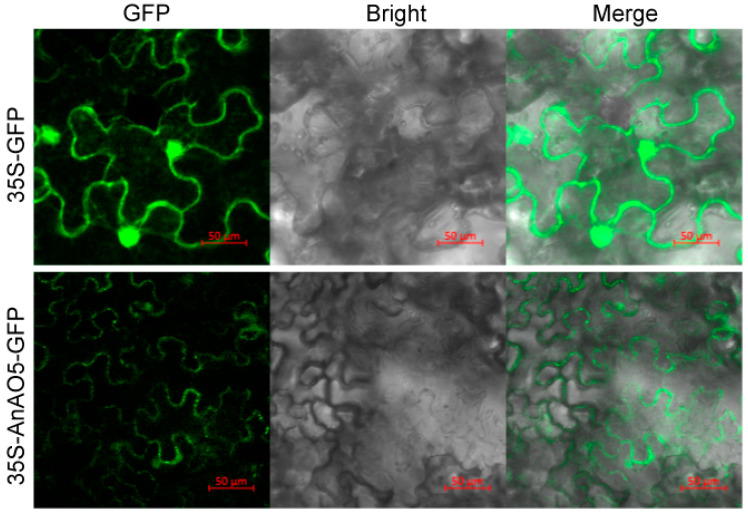
Subcellular localization analysis of AnAO5 protein by transiently expressing the *AnAO5* gene in tobacco. Bars = 50 μm.

The AnAO5 protein was predicted to be localized in apoplast. To experimentally determine the subcellular localization of *A. nanus* AnAO5, the AnAO5 gene was transiently expressed in tobacco leaves. The GFP florescence signal was detected in the intercellular space, supporting the apoplast localization of the AnAO5 protein (Figure 10).

We extracted apoplast fluid using the infiltration-centrifugation method, and measured the activities of MDH and AO. There was no MDH activity detected in the apoplast fluid, indicating no cytoplasmic contamination in the apoplast fluid. The ascorbate oxidase activity of the apoplast fluid was 45.68 ± 7.61 U/g*protein, indicating that the AnAO5 protein was localized in the apoplast in *A. nanus*.

### 2.11. Overexpression of AnAO5 Enhanced the Tolerance to Low-Temperature Stress in Yeast and Tobacco

**Figure 11:**
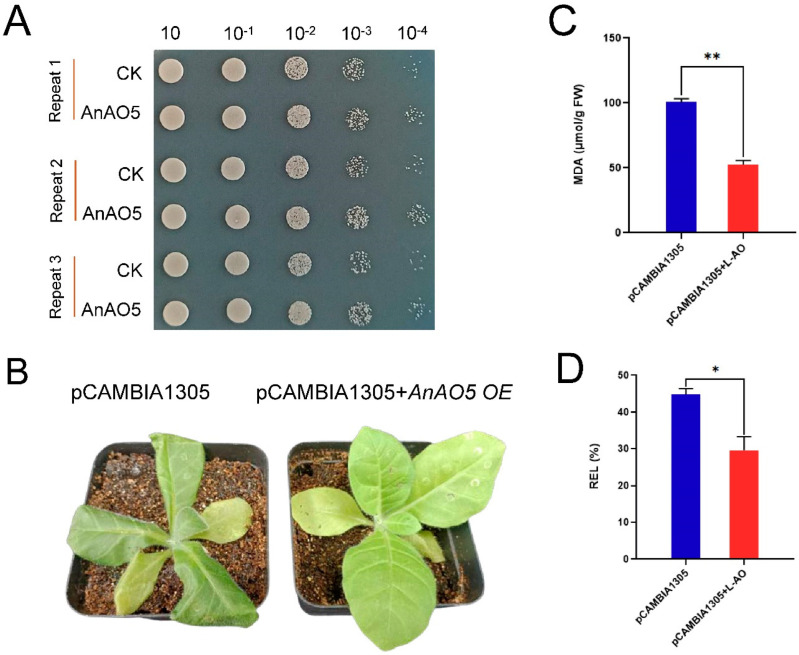
Overexpression of the *AnAO5* gene enhanced the tolerance to low temperature in yeast and tobacco seedlings. (**A**) Overexpression of *AnAO5* gene enhanced the tolerance to repeated freeze–thaw stress in yeast. (**B**) Tobacco seedlings transiently expressing the empty vector (control) and tobacco seedlings transiently expressing *AnAO5* gene were transferred to a −4 °C plant incubator for low temperature treatment for 24 h. (**C**) The MDA content of tobacco leaves after low-temperature treatment. (**D**) The values of REL of tobacco leaves after low-temperature treatment. Values are expressed as means ± SD (*n* = 3), and the statistically significant differences were evaluated using Student’s t-test, *, *p* < 0.05, **, *p* < 0.01.

To reveal the function of AnAO5 in low-temperature tolerance in yeast and tobacco, a yeast growth assay and analysis of transient expression of foreign genes in tobacco were performed. After repeated freeze–thaw treatments, the survival rate of the yeast-expressing *AnAO5* gene was significantly higher than the yeast transformed with pYES2 empty plasmid (Figure 11A), indicating that the expression of *AnAO5* gene alleviated the damage caused by repeated freeze–thaw stress in yeast.

After being cultured at −4 °C for 24 h, the tobacco transiently expressing the empty vector (pCAMBIA1305-GFP) exhibited obvious wilting, while no significant wilt symptom was observed in the tobacco seedlings transiently expressing the *AnAO5* gene (Figure 11B). Furthermore, compared with the tobacco transiently expressing the empty vector (pCAMBIA1305-GFP), less cell damage, as revealed by the malondialdehyde (MDA) and relative electrolyte leakage (REL) measurements (Figure 11C,D), was detected in the tobacco transiently expressing the *AnAO5* gene. These results showed that overexpression of *AnAO5* gene enhanced the tolerance to low-temperature stress in yeast and tobacco.

### 2.12. Overexpression of AnAO5 Gene Enhanced the Tolerance of A. thaliana to Cold Stress

**Figure 12:**
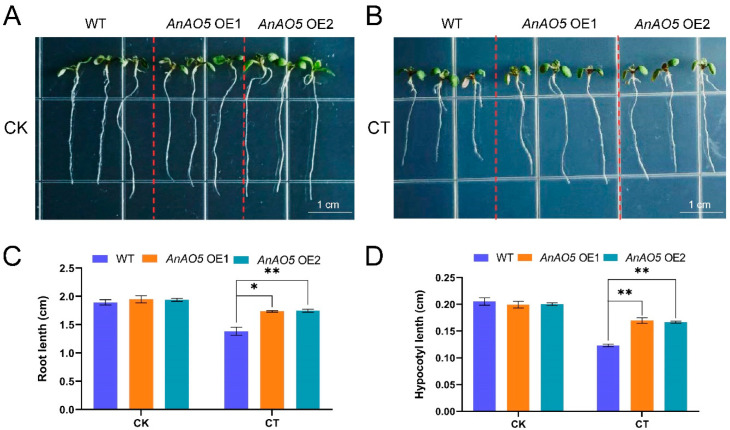
Overexpression of *AnAO5* enhanced the tolerance of Arabidopsis seedlings to low temperature. (**A**) Arabidopsis seedlings of wild-type, AnAO5 OE1, and AnAO5 OE2 cultured at room temperature (22 °C). (**B**) Arabidopsis seedlings of wild-type, AnAO5 OE1, and AnAO5 OE2 cultured under cold stress (15/10 °C). (**C**) The root lengths of Arabidopsis seedlings of wild-type, AnAO5 OE1, and AnAO5 OE2 in CK and CT. (**D**) The hypocotyl lengths of Arabidopsis seedlings of wild-type, AnAO5 OE1, and AnAO5 OE2 in CK and CT. Values are expressed as means ± SD (*n* = 3), and statistically significant differences were evaluated using Student’s t-test, * *p* < 0.05, ** *p* < 0.01.

To further evaluate the function of AnAO5 in plant cold stress tolerance, the *AnAO5* gene was introduced into Arabidopsis, and then the phenotypic differences between wild-type and two transgenic lines (AnAO5 OE1 and AnAO5 OE2) under room temperature (22 °C) and low temperature (15/10 °C) were analyzed. At room temperature (22 °C), the plants of the wild-type and the two transgenic lines exhibited similar growth performance (Figure 12A); Under cold stress, the roots and hypocotyls of wild-type plants and the transgenic lines (OE1 and OE2) were shorter than those of the seedlings grown under room temperature; however, compared with the wild type plants, the roots and hypocotyls of the transgenic lines (OE1 and OE2) were longer (Figure 12B–D). These results indicated that overexpression of the *AnAO5* gene enhanced the tolerance to cold stress in Arabidopsis.

## 3. Discussion

The uplift of the Qinghai-Tibet Plateau and the change in the Asian ocean monsoon led to the current cold and dry climate in Central Asia, as well as the related desert expansion, which has affected the geographical distribution, genetic diversity, lineage differentiation, and species formation of Tertiary relict plants [2]. The dramatic climate change caused by the uplift of the Qinghai-Tibet Plateau has also led to a great change in the habitat of plants in Central Asia [3]. The study of the Tertiary relict plants that survived after this dramatic change is of great significance, to understand the impact of long-term climate change on vegetation in Central Asia. The Tertiary relict plant *A. nanus* is a rare evergreen broad-leaved shrub in the desert region of Central Asia, which has high tolerance to environmental stresses such as drought and low temperature. Studying the gene family structure and evolutionary characteristics of *A. nanus* is helpful for understanding the response of plant genome evolution with environmental changes, and to understand the molecular mechanism associated with the adaptive evolution of desert plants.

The amino acid lengths and predicted molecular weights of the 16 AO members were not significantly different. Multiple sequence alignment showed that all AOs contained three conserved domains of ascorbate oxidase (Pfam gene models: PF00394, PF07731, and PF07732) [37] and also contained a transmembrane domain at the N-terminal. MEME analysis also showed that multiple motifs were detected in all AO members, and these motifs were motif 1, motif 2, motif 3, motif 6, motif 8, and motif 10. These data indicated that the AnAO family members are similar in structure.

However, there are also some differences in the structure of AO family members. Motif 4, and motif 5, and motif 9 were not detected in AnAO5, AnAO11, and AnAO15. There were three gaps in AnAO5, AnAO11, and AnAO15, and the first was a gap of three amino acid residues in the amino acids sequence corresponding to Pfam00394, the second was a gap of five amino acid residues in the amino acids sequence corresponding to Pfam07731, and the third was a gap of seven amino acid residues between the two gaps mentioned above.

Phylogenetic analysis clustered all AO members into four major clades, and the AO members in each clade had similar amino acid sequences. Corresponding to the structural difference discussed above, AnAO5, AnAO11, AnAO15, and Arabidopsis At4g39830 were clustered into an independent branch in the phylogenetic tree, indicating that the four AOs shared a common ancestor and perhaps had similar biological functions.

Gene duplication is one of the main forces driving expansion of gene families and we found that segmental duplication and tandem duplication were the main ways to form the AO gene family of *A. nanus*. In the present study, there were six AO gene pairs that underwent segmental duplication and two AO gene pairs that underwent tandem duplication. According to the Ks distribution curves of the gene pairs undergoing segmental duplication, we could infer the time of WGD events leading to segmental duplication of AO genes. The four *AnAO* genes, i.e., *AnAO1*, *AnAO9*, *AnAO14*, and *AnAO16* formed four pairs of segmental duplication gene pairs, indicating that they shared a common ancestor. In the phylogenetic tree, these four AO genes were located in the same clade, supporting their common ancestry. These four AOs were generated from their common ancestor by two WGD events, and the most recent occurred about 85.37 Mya, resulting in the differentiation of AnAO1–AnAO16 and AnAO9–AnAO14. In the phylogenetic tree, AnAO1 and AnAO16, and AnAO9 and AnAO14 were clustered into two independent branches, respectively, which supports the above speculation.

Generally speaking, the number of orthologous gene pairs among species is positively correlated with their relationship. In the present study, there were 6, 7, 7, 11, and 18 orthologous gene pairs between *A. nanus* and *V. vinifera*, *A. thaliana*, *M. truncatula*, *T. pratense*, and *L. albus*, respectively. Of the 5 plant species used for collinearity analysis, *L. albus* was the closest species to *A. nanus*, and the number of orthologous gene pairs between the two species was the largest.

In previous studies, we proved that miR4415 was a legume-specific miRNA, which may have evolved independently after the emergence of a common ancestor of leguminous plants [38]. Interestingly, an AO-targeting miRNA, miR528, was also identified in rice, which was proven to be a monocotyledonous plant-specific miRNA [33], and the rice *AO* gene targeted by miR528 is an ortholog of *AnAO5* (Figure 8A). Considering that miR4415 and miR528, the two miRNAs targeting *AO* genes evolved independently from leguminous plants and monocotyledonous plants, we speculated that, compared with other *AO* genes in *A. nanus*, miRNA-targeted *AO* genes, i.e., *AnAO5* and *AnAO11*, may play more important biological roles in plant growth and development, as well as environmental stress response, in *A. nanus*.

Why are AnAO5, AnAO11, and AnAO15 closely related in evolution, and yet the *AnAO15* gene is not the target of miR4415? The first possibility is that the binding of miR4415 to *AnAO5* gene and *AnAO11* gene evolved after the common ancestor of *AnAO5* and *AnAO11* appeared. Another possibility is that the binding of miR4415 to *AnAO5* gene and *AnAO11* gene evolved after the emergence of common ancestors of *AnAO5*, *AnAO11*, and *AnAO15*, but later the mutation occurred at the binding site of miR4415 to *AnAO15* gene, resulting in the loss of the binding ability of miR4415 to *AnAO15* gene.

Promoter analysis showed that cis-acting elements involved in drought, low-temperature, and ABA responses were distributed on the promoters of most *AnAO* genes, indicating that *AnAO* genes might participate in drought and low temperature stress responses. Transcriptome data and qRT-PCR analysis were used to reveal the expression of *AnAO* genes in different tissues and under osmotic and cold stress. The responses of *AnAO* gene expression to osmotic stress and cold stress were different. All *AnAO* genes were downregulated under osmotic stress for 1 d and 7 d. Under cold stress of 1 d, all *AnAO* genes were downregulated, but three *AnAO* genes, i.e., *AnAO5*, *AnAO13*, and *AnAO16*, were upregulated under cold stress of 7 d. The increased expression of *AnAO5*, *AnAO13*, and *AnAO16* coincided with the increase of the AO activity under cold stress of 7 d, implying the three *AnAO* genes contributed to the increase of the AO activity under cold stress of 7 d and played important roles in the cold adaptation of *A. nanus*.

There are three reasons why we choose the *AnAO5* genes for further functional analysis. First, the expression level of *AnAO5* gene was downregulated after 1 d of cold stress and upregulated after 7 d of cold stress. Second, there were five cis-acting elements involved in ABA response, three cis-acting elements involved in gibberellin response, one cis-acting elements involved in drought response, and one cis-acting elements involved in low-temperature response in the promoter region of *AnAO5* gene, indicating that *AnAO5* gene might play an important role in plant response to low-temperature and drought stress. Third, *AnAO5* was one of the two *AnAO* genes targeted by *miR4415*.

Multiple sequence alignment analysis showed that AnAO5 contained the three Pfam gene models for ascorbate oxidase and a transmembrane domain at the N-terminal. The subcellular localization study showed that AnAO5 was located in the apoplast [38]. The ortholog of AnAO5 in Arabidopsis is At4g39830, which was reported to encoded an apoplast localized AO. At4g39830 was reported to be involved in plant defense against insects [39], and play a role in the regulation of apoplastic iron excess [40]. The ortholog of AnAO5 in rice is OsAAO1, which encoded an apoplast localized AO and was targeted by miR528. OsAAO1 was involved in the response to high salinity stress, and inhibition of OsAAO1 expression led to increased tolerance to salt stress in rice [33]. The functional study of *AnAO5* orthologous genes in other plants, and the existence of multiple stress and hormone response cis-acting elements on the *AnAO5* promoter, suggested that AnAO5 may play an important role in the stress response of *A. nanus*.

Based on the response patterns of AnAO5 gene expression and AO enzyme activity to low-temperature and drought stresses, we expressed *AnAO5* gene in yeast, tobacco, and Arabidopsis, and focused on the effect of *AnAO5* gene on the tolerance of cells and plants to low-temperature stress. Yeast cells expressing *AnAO5* gene had a higher survival rate under repeated freeze–thaw stress, and tobacco transiently expressing *AnAO5* gene showed high tolerance to short-term low-temperature stress. Compared with the wild type, the root and hypocotyl growth rate of two Arabidopsis lines overexpressing *AnAO5* gene was relatively fast under low-temperature conditions, showing a high tolerance to low-temperature stress. These results indicated that overexpression of *AnAO5* gene enhanced the tolerance of plants to low temperature and that *AnAO5* may be involved in cold acclimation in *A. nanus*.

The expression pattern of *AnAO5* was consistent with the change pattern of the total AO enzyme activity in *A. nanus* under low temperature, suggesting that AnAO5 protein may be one of the main contributors to AO enzyme activity in *A. nanus*. As an apoplast-located AO, AnAO5 may participate in the response of plants to environmental stress and play other possible biological functions by regulating the redox balance of the apoplast via catalyzing the oxidation of AsA. In cold-acclimated *A. nanus*, the upregulation expression of *AnAO5* gene led to decreased ASA content and increased DHA content, finally lowering the AsA/DHA ratio in apoplast, which was conducive to the accumulation of ROS [38]. It was reported that ROS, including H_2_O_2_ and hydroxyl radicals, was involved in plant growth, development, and the response to environmental stress and, as a signal molecule, H_2_O_2_ affected a variety of biological processes, including inducing stomatal closure, further enhancing the tolerance of plants to adverse external environments [14,17]. It was observed that tobacco plants overexpressing AO gene had higher levels of H_2_O_2_ [25]. Thus, it is speculated that AnAO5 might contribute to the tolerance of cold stress by regulating the apoplastic redox state in *A. nanus*.

## 4. Materials and Methods

### 4.1. Identification of the AO Proteins in A. nanus

The genomic sequences and related information of *A. nanus* were downloaded from GigaScience Database [8,41]. The genomes of *A. thaliana*, *O. sativa*, *V. vinifera*, *M. truncatula*, *G. max*, *T. pratense*, and *L. albus* were downloaded from the NCBI database. HMMER3 software was used to identify the AO family members in *A. nanus* [42], based on the AO gene model (PF00394, PF07731, and PF07732) in the Pfam database. All candidate sequences were manually checked using the HMMER web server (https://www.ebi.ac.uk/Tools/hmmer/ (accessed on 30 August 2022)). The physicochemical properties of AO were predicted using the ProtParam tool (http://web.expasy.org/protparam/ (accessed on 30 August 2022)) [43]. Subcellular localization was predicted using the WoLF PSORT tool [44].

### 4.2. Chromosomal Location and Gene Structure Analysis of A. nanus AO Family Genes

The location of *AnAO* genes on chromosomes and the exon-intron distribution of AnAO were visualized using TBtools software [45]. The conserved motifs of AnAO proteins were identified using the MEME website (Multiple Expectation Maximization for Motif Elicitation) (http://meme-suite.org/ (accessed on 30 August 2022)) [46].

### 4.3. Multiple Sequence Alignment and Phylogenetic Analysis

Multiple sequence alignment was performed using the MUCSLE algorithm [47]. The phylogenetic tree was constructed using MEGA 7 [48], and bootstrap analysis was conducted using 1000 replicates. The synteny analysis of *AnAO* genes was performed using MCScanX tools [49]. The synonymous substitution rate (Ks), nonsynonymous substitution rate (Ka), and Ka/Ks ratio between homologous gene pairs were calculated using KaKs_Calculator 2.0 [50].

The base evolutionary rate (λ) of *A. nanus* was calculated using the formula λ = Ks/2T, with Ks indicating the synonymous substitution rate in *A. nanus*, and T indicating the time of divergence between the two species, which was queried through the TIMETREE website (http://www.timetree.org/ (accessed on 30 August 2022)). The duplication time of homologous genes within the *A. nanus* gene family was calculated using the formula T = Ks/2λ.

### 4.4. Prediction of Cis-Acting Elements in the Promoter Regions of AnAO Genes

The PlantCARE database (https://bioinformatics.psb.ugent.be/webtools/plantcare/html/ (accessed on 30 August 2022)) [51] was used to predict the cis-acting elements in the 2000 bp promoter region upstream of each AnAO gene’s start codon, and the results were visualized using TBtools software [45].

### 4.5. Gene Expression Analysis Based on the Transcriptome Data

Twelve transcriptomic datasets of *A. nanus* were downloaded from the SRA database with the accession numbers SRR11089024-SRR11089029 and SRR11087599-SRR11087604, which contain transcriptome data from the control group, osmotic treatment group (20% PEG6000 solution for 7 d), cold stress treatment group (4 °C for 7 d), and *A. nanus* leaves in spring and winter. The gene expression level of each gene was calculated using the Kallisto quant [52].

### 4.6. Plant Materials, Stress Treatment, and AO Activity Analysis

The seeds of *A. nanus* were collected from Wuqia county, Xinjiang autonomous district, China. The seed germination and planting conditions of *A. nanus* were based on a previous study [53]. Osmotic and cold stress treatments were performed with reference to a previous study [54]. In brief, seedlings of *A. nanus* were randomly divided into 11 groups, and one group was grown in normal conditions and was used as the control group. The five osmotic stress treatment groups were irrigated with 20% PEG6000 for 3 h, 6 h, 12 h, 24 h, and 7 d. The other five groups were transferred to a 4 °C plant incubator for cold stress treatment for 3 h, 6 h, 12 h, 24 h, and 7 d. Leaf samples from the control groups and the treatments group were collected and snap-frozen in liquid nitrogen, then the samples were stored at −80 °C until RNA extraction.

The leaf apoplast fluid was obtained using the infiltration-centrifugation method [55]. Water was used as the infiltration buffer, a 60 mL syringe was used for leaf infiltration, and the resulting leaf apoplast fluid was recovered by centrifugation for 10 min at 3000 g. The activities of AO were measured using kits manufactured by Solar-bio Science & Technology Co., Ltd. (Beijing, China).

### 4.7. RNA Extraction and Quantitative Real-Time PCR (qRT-PCR) Analysis

Total RNA was extracted from the leaves of *A. nanus* using the Trizol reagent, following the manufacturer’s directions (Invitrogen, Waltham, MA, USA), and reverse transcription was conducted using a FastQuant RT Kit (TIANGEN, Beijing, China). qRT-PCR analysis was performed according to the methods described previously [56], and *eukaryotic translation initiation factor 1 (eIF1)* gene was used as the internal control. The primers used for qRT-PCR are listed in Supplementary Table S1. Three biological replicates were used for each group and three technical replicates of each biological replicate were analyzed. The relative expression of the genes was calculated using the 2^−ΔΔCt^ method [57].

### 4.8. Vector Construction and Expression in Yeast

The *AnAO5* gene was expressed in yeast according to a previous method [58]. *AnAO5* gene was inserted into the pYES2 vector using the restriction sites of *Bam*HI and *Hind*III, and then the recombinant plasmid was transferred into the INVSCL yeast strain. When the yeast was in logarithmic phase, the culture medium was transferred to −20 refrigerator for freezing treatment. Yeast was frozen at −20 °C for 1 h and then thawed at room temperature, and the treatment was repeated three times. Finally, the freezing tolerance of yeast was analyzed by counting the number of colonies on SD-Ura solid medium.

### 4.9. Transient Transformation of AnAO5 in Tobacco and the Subcellular Locations

Tobacco seeds were sown in peat soil and vermiculite matrix at a fully mixed volume ratio of 1:1. The seedlings were cultured in a greenhouse at 25 °C, with a light intensity of 400 μmol·m^−2^·s^−1^ and a photoperiod of 16/8 h (light/dark). Six seedlings with similar growth status were selected and divided into two groups. The AnAO5 was transiently expressed in tobacco by reference to a previous method [59]. The vector used in the experiment was pCAMBIA1305, the enzyme digestion sites were *Xba*I and *Bam*HI, and the competent cell was *Agrobacterium tumefaciens* GV3101. One group of tobacco plants was transformed transiently with pCAMBIA1305 empty vector, and the other group of plants was transformed transiently with pCAMBIA1305 vector ligated with AnAO5. All tobacco plants were transferred to a −4 °C plant incubator for cold stress treatment for 24 h. After low-temperature treatment, the growth state of tobacco was observed. MDA and REL were measured according to a previously described method [60]. The subcellular locations of the AnAO5 were imaged using an OLYMPUS Inverted Fluorescence Microscope IX81 [61].

### 4.10. Transformation of AnAO5 in Arabidopsis and Evaluation of Low-Temperature Tolerance

The CDS of *AnAO5* gene was ligated into the vector of pCAMBIA1305, the enzyme digestion sites were *Xba*I and *Bam*HI, and the competent cell was *A. tumefaciens* GV3101. The constructs were transformed into Arabidopsis Columbia-0 via the floral dip method [62]. Transgenic plants were selected using 25 μg/mL hygromycin. Seeds of wild-type and two transgenic lines were surface-sterilized and sown on Murashige and Skoog (MS) agar plates. Plants were grown in a greenhouse at 22 °C, with a light intensity of 400 μmol·m^−2^·s^−1^ and a photoperiod of 16/8 h (light/dark). Seven-day-old seedlings grown under normal conditions were randomly divided into 2 groups. The control group (CK) of seedlings were was at room temperature (22 °C) for 7 d, and the cold treatment group (CT) was kept at low temperature (15/10 °C) for 7 d. The root length and hypocotyl length of WT and transgenic plants in the CK and CT groups were measured for evaluation of low-temperature tolerance.

## Figures and Tables

**Table 1:** Characterization of predicted AOs in *A. nanus*.

Gene ID	Gene Name	Length (aa)	MW (kD)	pI	Instability Index	Aliphatic Index	GRAVY	Subcellular Location *
EVM0035481.1	AnAO1	544	61.43	9.67	45.34	77.21	−0.29	cyto
EVM0014986.1	AnAO2	539	60.17	8.65	33.59	90.43	−0.21	chlo
EVM0002929.1	AnAO3	566	63.90	8.23	35.45	82.46	−0.28	extr
EVM0000882.1	AnAO4	567	63.75	8.81	36.68	84.23	−0.24	plas
EVM0030483.1	AnAO5	573	64.33	8.69	34.57	81.01	−0.28	extr
EVM0001519.1	AnAO6	540	60.25	9.24	33.9	89.56	−0.17	vacu
EVM0025631.1	AnAO7	539	60.28	9.05	37.2	87.51	−0.24	chlo
EVM0000071.1	AnAO8	544	60.33	8.71	28.79	84.89	−0.23	extr
EVM0006597.1	AnAO9	538	59.61	5.76	46.35	82.04	−0.22	vacu
EVM0023147.1	AnAO10	551	61.65	8.66	33.82	87.89	−0.18	vacu
EVM0022092.1	AnAO11	574	64.33	8.24	35.87	80.68	−0.31	vacu
EVM0018204.1	AnAO12	547	60.89	9.53	33.75	86.95	−0.24	nucl
EVM0008086.1	AnAO13	548	61.44	8.91	36.00	84.51	−0.23	vacu
EVM0022681.1	AnAO14	542	60.46	8.75	44.28	85.59	−0.21	vacu
EVM0005397.1	AnAO15	577	65.22	8.68	40.73	77.68	−0.34	extr
EVM0034083.1	AnAO16	517	58.42	9.82	44.6	79.73	−0.27	chlo

* extr: extracellular matrix, chlo: chloroplast, plas: plasma membrane.

**Table 2:** Analysis of evolutionary selection pressure on the AO gene family of *A. nanus*.

Paralogous Pairs	Ka	Ks	Ka/Ks	Type
AnAO1–AnAO14	0.195	1.125	0.173	Segmental Duplication
AnAO1–AnAO9	0.207	1.485	0.139	Segmental Duplication
AnAO2–AnAO6	0.252	1.796	0.140	Segmental Duplication
AnAO12–AnAO8	0.043	0.355	0.121	Segmental Duplication
AnAO14–AnAO9	0.055	0.339	0.162	Segmental Duplication
AnAO16–AnAO1	0.068	0.299	0.226	Segmental Duplication
AnAO3–AnAO4	0.141	1.022	0.138	Tandem Duplication
AnAO6–AnAO7	0.277	1.467	0.189	Tandem Duplication

## Data Availability

No new data were created or analyzed in this study. Data sharing is not applicable to this article.

## Supplementary Materials

The following supporting information can be downloaded at: https://www.mdpi.com/article/10.3390/plants12030677/s1. Figure S1: Ks distribution curves of AO genes undergone segmental duplication; Table S1: A list of fluorescent quantitative and vector construction primers.
