# Supplementary figures and images for: Gene Profiling of the Ascorbate Oxidase Family Genes under Osmotic and Cold Stress Reveals the Role of AnAO5 in Cold Adaptation in Ammopiptanthus nanus
Source: Plants (Basel). 2023 Feb 3;12(3):677. doi: 10.3390/plants12030677 (PMC9920380; doi:10.3390/plants12030677)

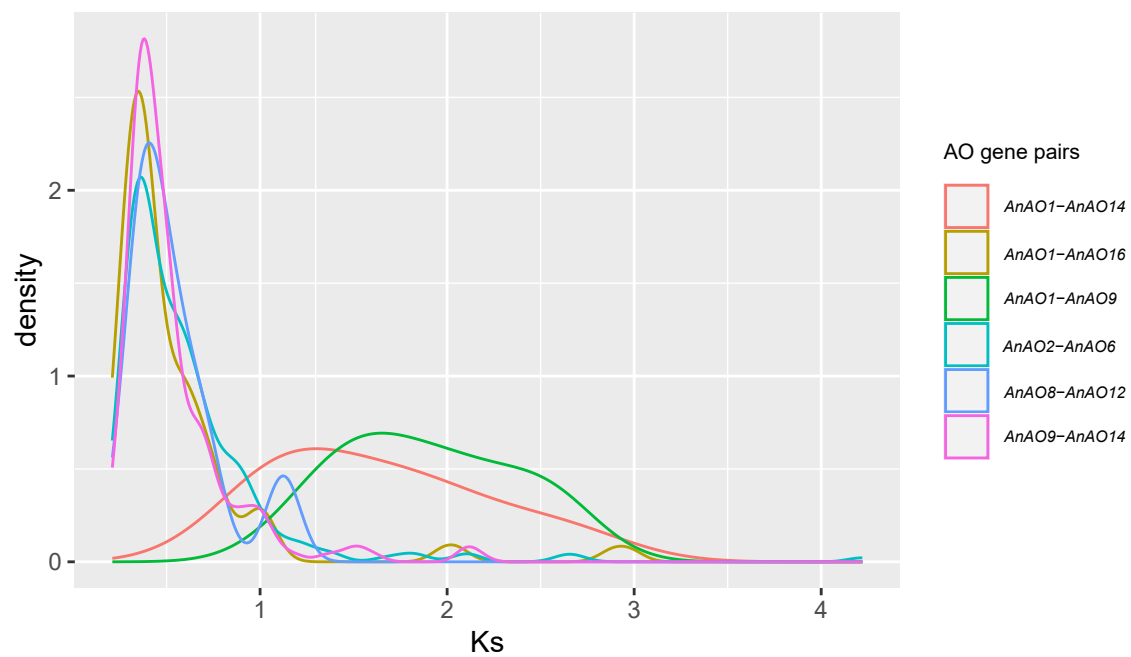

Supplement: Supplementary file 1 [file plants-12-00677-s001.zip › Figure_S1_Ks distribution curves of AO genes undergone segmental duplication.pdf]
